# Medical cannabis for the management of pain in chronic pancreatitis with recurrent exacerbations: a case report

**DOI:** 10.1186/s42238-025-00303-w

**Published:** 2025-08-08

**Authors:** Felice Antonio Spaccavento, Cesare De Virgilio Suglia, Silvio Tafuri, Angela De Trizio, Rossella Giannuzzi, Filomena Cavallera, Fabio Turco

**Affiliations:** 1 Palliative Care Unit, ASL Bari (Bari Local Health Trust), Bari, Italy; 2 Department of Precision and Regenerative Medicine and Ionian Area (DiMePreJ), University of Bari Aldo Moro, Bari, Italy; 3 Cannabiscientia SA, Lugano, Switzerland; 4 Altamura Territorial Pharmacy (Bari Local Health Trust), Altamura, Italy

**Keywords:** Pancreatitis, Medical Cannabis, Pain management, Cannabidiol, Tetrahydrocannabinol

## Abstract

**Introduction:**

Control of pain in patients affected by chronic pancreatitis with recurrent exacerbations is a challenging condition, with conventional therapies often providing limited relief. This case report describes the use of medical cannabis as a novel approach in a patient with refractory chronic pancreatitis, contributing to the growing interest in alternative treatments for pain and inflammation in similar complex cases.

**Case presentation:**

A 54-year-old woman with a 24-year history of chronic pancreatitis caused by recurrent acute pancreatitis presented with persistent, severe abdominal pain and recurrent exacerbations despite undergoing numerous conventional interventions, including cholecystectomy, enzyme supplementation, repeated endoscopic retrograde cholangiopancreatographies (ERCPs), and stent placements. Imaging and laboratory findings confirmed chronic pancreatitis, with evidence of Oddi sphincter stenosis and microlithiasis. The patient was initially managed with standard pain relief therapy, digestive enzymes, and endoscopic interventions, all of which failed to provide lasting relief. In February 2024, she began treatment with a medical cannabis formulation rich in Cannabidiol, under the supervision of her healthcare provider. This intervention led to substantial pain reduction, cessation of acute episodes, improved appetite, and enhanced quality of life.

**Conclusion:**

This case illustrates that medical cannabis may offer a promising alternative for managing chronic pancreatitis, especially when conventional treatments prove ineffective. This outcome underscores the need for further research on cannabinoids as a therapeutic option in chronic pain and inflammation management for pancreatitis and other challenging conditions.

## Introduction

Acute pancreatitis (AP) is a sudden inflammation of the pancreas, manifesting as acute abdominal pain and elevated pancreatic enzymes serum titer: clinical presentation could range from mild to severe. (Szatmary et al. 2022) Severe AP often leads to significant complications and a high case fatality rate. Managing AP traditionally involves fasting, fluid resuscitation, pain management, and addressing underlying causes (García-Rayado et al. 2020; Quinlan 2014). However, chronic and recurrent cases are challenging, often requiring ongoing interventions due to persistent symptoms and exacerbations. The treatment of pain in chronic pancreatitis represents a major challenge for which many analgesic drugs has been used in the past, without an universal agreement on the ideal analgesic regime, as common to many chronic pain syndromes (Kuhlmann et al. 2025).

The potential role of medical cannabis in managing chronic pain conditions, including pancreatitis, has gained interest in recent years (Maharajan et al. 2020). Cannabinoids—particularly tetrahydrocannabinol (THC) and cannabidiol (CBD)—interact with the body’s endocannabinoid system, primarily through CB1 and CB2 receptors (Stella 2023; Lu and MacKie 2015). These receptors are distributed widely in the body, including in pancreatic tissue, where they influence pain perception and inflammatory responses. THC, the primary psychoactive compound in cannabis, has been linked to pain modulation and may promote pancreatic fibrosis, while CBD, a non-psychoactive component, demonstrates significant anti-inflammatory properties and could reduce inflammation and modulate immune responses in chronic conditions (Chayasirisobhon 2020).

In Italy, the use of cannabis for therapeutic purposes is regulated and is establishing itself as an option for various conditions, in particular for the management of chronic pain. Among the pathologies for which it is often prescribed, in addition to chronic pain (which includes oncological pain, neuropathic pain, pain from spasticity, pain refractory to other therapies), we find: Multiple Sclerosis, spinal cord injuries, nausea and vomiting from chemotherapy, glaucoma, Tourette's syndrome, fibromyalgia.

This case represents a first-report of a patient with a long history of recurrent acute pancreatitis who experienced substantial symptom relief after introducing medical cannabis. Her response suggests medical cannabis may offer a novel and effective approach for chronic and treatment-resistant pancreatitis, contributing valuable insights for further research and therapeutic strategies.

## Case presentation

The patient is a 54-year-old woman with a 24-year history of recurrent acute pancreatitis, first diagnosed following a severe episode at age 25. Her primary symptoms included intense, postprandial abdominal pain, often misattributed to renal colic in the early years, and epigastric pain with retrosternal irradiation, to which were frequently added vomiting and sensations of nausea. She has an extensive medical history of chronic pancreatitis with recurrent exacerbations, with several hospitalization episodes. Her family history is unremarkable for pancreatic or related gastrointestinal conditions, and she has no known genetic predispositions associated with pancreatitis. She has never been a smoker and totally abstains from the consumption of alcoholic beverages.

Over the years, the patient underwent various diagnostic evaluations and interventions, including multiple endoscopic retrograde cholangiopancreatographies (ERCPs) with biliary and pancreatic sphincterotomies, removal of stones through Fogarty catheter balloon and placement of endopancreatic prostheses to dilate the pancreatic duct after the countless cases of obstruction and consequent Post-ERCP Acute Pancreatitis (PEAP). Among the other tests she was subjected to we can mention esophagogastroduodenoscopies (EGDs), biliopancreatic ultrasound endoscopy, and imaging studies like CT and MRI, which confirmed features compatible with chronic pancreatitis. Despite surgical interventions, including cholecystectomy, sphincterotomies and several stent placements, her symptoms persisted, impacting her quality of life significantly. Psychosocially, the chronic pain and frequent hospitalizations contributed to anxiety and decreased appetite, leading to weight loss up to weighing 36 kg (BMI 15) and diminished overall well-being.

### Clinical findings

On physical examination, the patient presented with marked abdominal tenderness, particularly in the epigastric retrosternal region, consistent with her recurrent episodes of pancreatitis. Notably, during acute exacerbations, she exhibited guarding and tenderness without signs of peritonitis. Her vital signs during these episodes remained stable, although the severity of pain significantly impaired her daily functioning to the point of making her underweight due to lack of appetite (BMI 15).

Laboratory tests frequently showed elevated serum amylase and lipase levels, aligning with her acute pancreatitis diagnoses. Imaging studies, including computed tomography (CT) and magnetic resonance cholangiopancreatography (MRCP), consistently revealed pancreatic inflammation and structural changes associated with chronic pancreatitis characterized by pancreas with swollen appearance, edematous imbibition, fibrotic tracts that give poor representation of the lobulation, irregular dilation of the pancreatic duct and stenotic tracts at the level of the head and tail. ERCP confirmed these findings and further documented the recurrent common bile duct microlithiasis, which was suspected as a contributing factor to her recurrent symptoms.

### Timeline

- 1995: first episode of severe abdominal pain, leading to diagnosis of enterocolitis and edematous pancreatitis.
- 1995—2003: Multiple episodes of severe abdominal pain, initially referred to a possible renal colic.
- 2003: Hospitalized at the Polyclinic of Bari, Italy, where a diagnosis of acute pancreatitis was formulated, on a background of chronic recurrent pancreatitis after EGD, CT, and ERCP evaluations. First ERCP procedure performed.
- July 2003: Admitted to hospital in Verona, Italy, for severe acute pancreatitis and hypokalemia. Underwent laparoscopic cholecystectomy due to microlithiasis.
- 2004—2007: several hospitalization episodes for recurrent pancreatitis. She was subjected to multiple EGDs, colonoscopies, MRCPs, and repeated ERCP with with biliary and pancreatic sphincterotomy, along with several stent placements and removals consequently to Post-ERCP Acute Pancreatitis.
- 2008—2010: new hospitalization episodes in various Italian hospitals, for abdominal pain and pancreatitis. Treated for biliary colic, chronic gastritis with H. pylori, and chronic pancreatitis with cicatricial stenosis of the sphincter of Oddi and the papillary area, and dilation of the Wirsung duct.
- 2011—2016: she reported severe pain and recurrent pancreatitis episodes. Underwent further ERCPs with sphincterotomy enlargement, stent placements, Fogarty balloon stone removal, and laparoscopic procedures across several hospitals.
- 2017–2020: Progressive deterioration with frequent hospital admission for severe abdominal pain and pancreatitis exacerbations due to recurrent obstructions of the pancreatic endoprosthesis at the level of the main duct.
- September—October 2023: Hospitalized for acute exacerbations of pancreatitis; underwent additional ERCP and stent placement without significant improvement.
- February 2024: Initiated on medical cannabis for pain management at the Pain Therapy Clinic at ASL Bari, Italy, leading to significant symptom relief and cessation of colic episodes.
- January 2025: The patient comes to the outpatient pain clinic for the scheduled checkup in the planned follow-up year. Pain has ceased completely, no flare-ups in the past 11 months, no need for hospitalization or need for additional pain medication other than cannabis extract
- June 2025: the patient comes back for the last visit. Confirms the total absence of pain, the ultrasound and blood tests show no alterations, the weight gain has brought her to a normal weight Body Mass Index (BMI 20, before the treatment it was 15)

### Therapeutic intervention

The patient’s treatment history includes numerous interventions aimed at managing her chronic pancreatitis and alleviating her recurrent pain. Initially, she was treated with traditional approaches, including fasting, intravenous fluid resuscitation, and analgesics to manage acute episodes. In 2003, following a confirmed diagnosis of microlithiasis, she underwent a laparoscopic cholecystectomy; however, this procedure did not provide lasting relief, and her symptoms persisted. Over the following years, she was prescribed enzyme supplements, such as pancreatin-based supplements, to aid digestion, and her pain was managed with medications, including Butylscopolamine, Paracetamol and Ketorolac. Opioid painkillers had been suspended due to the patient's intolerance to these types of drugs (they cause severe confusion and drowsiness) and the risk of iatrogenic stenosis they entail.

Due to ongoing symptoms, she required multiple ERCP procedures, with associated sphincterotomies biliary and pancreatic and stent placements at the level of the pancreatic duct, aimed at relieving pancreatic ductal obstruction and recurrent cicatricial stenoses. Unfortunately, her condition remained resistant to these interventions, leading to repeated hospitalizations and additional procedures across various medical centers. Despite these extensive efforts, her abdominal pain and pancreatitis episodes continued unabated.

The option of major pancreatic surgery has always been deemed impracticable by the various hospitals she turned to, since the anatomical conformation of the patient's pancreas and her condition did not make it convenient from a risk–benefit point of view.

The refractory nature of her symptoms and limited response to traditional therapies led her medical team to consider alternative treatment options to address her persistent pain and inflammation.

In February 2024, after nearly two decades of unrelieved symptoms, the patient consulted the Pain Therapy Clinic at ASL Bari. Given the refractory nature of her condition, her medical team started treatment with a cannabis-based medicinal preparation consisting of a 5% cannabidiol (CBD) extract (6 ml), with < 0.5% of tetrahydrocannabinol (THC), diluted in a medium-chain triglyceride (MCT) oil, a pharmaceutical-grade lipophilic solvent, to a total volume of 50 ml, designed for precise dosing and therapeutic use aiming to control her severe pain. She was prescribed a regimen of 10 drops to be taken 3 times a day, equivalent to a total of 1 ml per day, for a total dosage of 6 mg of CBD per day. Remarkably, within days of starting this therapy, the patient experienced a dramatic reduction in pain and a cessation of acute pancreatitis episodes. This significant improvement allowed her to discontinue the use of other pain medications, while also contributing to enhanced appetite, weight gain, and an improved overall quality of life.

Prior to the initiation of cannabis therapy, for about 20 years, the patient experienced episodes of pain flare-ups 1–2 times a week: Epigastric pain radiating posteriorly, requiring immediate pain relief therapy, attributed through the visual-analog scale (VAS) to a pain score of 7–8. Pain 1–2 times a year became so intolerable (VAS score 9–10) that it could not be controlled by medication and required hospitalization. Even in the interval between colic episodes, the patient complained of chronic intermittent pain, a sensation of discomfort that did not interfere with daily activities but caused her inappetence, attributed to VAS score 2–3.

After the introduction of cannabis therapy, the patient reported a total absence of pain and a state of total well-being, attributing a VAS score of “0” on the VAS scale. This intervention with medical cannabis marked a turning point in her management, offering a level of symptom relief previously unattainable with conventional therapies.

After initiating cannabis treatment, the patient reported no adverse events. During subsequent follow-ups, we specifically inquired about symptoms such as drowsiness, mental confusion, orthostatic hypotension, and dizziness, but the patient consistently denied experiencing any of these effects.

### Follow-up and outcomes

Follow-up evaluation was carried out for 16 months (February 2024- June 2025).

Following the introduction of medical cannabis in February 2024, the patient’s condition showed a marked and sustained improvement. Routine follow-up appointments at the Pain Therapy Clinic, carried out each three months, confirmed that she experienced significant and lasting pain relief, with no further episodes of acute pancreatitis. Remarkably, the need for additional pain medications, including Ketorolac, Paracetamol and Butylscopolamine, was eliminated, as cannabis preparation effectively controlled her symptoms. The patient reported a notable improvement in her appetite, leading to healthy weight gain and enhanced energy levels. During the hospitalization period the patient had weighed less than 37 kg (BMI 15), but at the last check-up she had reached a normal weight of 58 kg (BMI 20).

Beyond pain relief, the intervention positively impacted other areas of her health. She reported a significant reduction in anxiety and an improvement in sleep quality, which allowed her to discontinue anxiety and sleep medications. Additionally, she resumed normal daily activities, something she had been unable to maintain for years due to chronic pain and frequent hospitalizations. A particularly unexpected outcome was the return of her menstrual cycle, which had been absent for over five years. Her gynecologist attributed this change to the overall improvement in her physiological state following the period of well-being induced by the cannabis treatment.

Following the initiation of treatment, no hospitalization episodes were reported. In contrast, during the five years prior to treatment, the patient experienced an average of 1–2 hospitalizations per year.

Overall, the patient’s quality of life improved substantially, marking a transformative shift in her health status after decades of refractory symptoms. The sustained relief observed with medical cannabis highlights its potential as an effective option for managing chronic and recurrent pancreatitis, especially in patients unresponsive to conventional therapies.

## Discussion

This case highlights the complex and challenging management of chronic pancreatitis caused by recurrent acute pancreatitis and the potential role of medical cannabis as a novel therapeutic option. The patient's extensive medical history demonstrates the limitations of conventional treatments, which often provide limited and temporary relief. Despite multiple ERCPs, sphincterotomies, stent placements, and various pain management strategies, the patient continued to suffer from frequent and severe episodes of pancreatitis.

The introduction of the cannabis-based medication, resulted in significant symptom relief, suggesting a potential new avenue for managing chronic pancreatitis. According to a study by Barlowe et al (2019), medical cannabis has shown efficacy in reducing opioid use and hospital visits in patients with chronic pancreatitis, highlighting its potential as a pain management strategy (Barlowe et al. 2019). This aligns with the observed benefits in our patient, who experienced a complete cessation of pain flare-ups and acute pancreatitis attacks, eliminating the need for additional pain medications like Ketorolac, Paracetamol and Butylscopolamine. However, contrasting evidence has identified cannabis use as a potential risk factor for acute pancreatitis, particularly in young adults, underscoring the importance of careful patient selection and monitoring when considering cannabis-based therapies (Barkin et al. 2017).

A strength of this case is the demonstrated efficacy of medical cannabis in a treatment-resistant patient, highlighting its potential for managing chronic and refractory pancreatitis. The patient's remarkable response supports the hypothesis that cannabinoids may reduce the frequency and severity of pancreatitis episodes, possibly due to their anti-inflammatory properties. Studies suggest that cannabinoids may exert protective effects in certain contexts, such as alcohol-associated pancreatitis, further supporting their therapeutic potential (Adejumo et al. 2018). This protective effect may be due to the anti-inflammatory properties of cannabinoids, which modulate the body's inflammatory response. Our patient's positive response to cannabis treatment supports this hypothesis, indicating that cannabis may help reduce the frequency and severity of pancreatitis episodes. This is in line with various systematic review and meta-analyses, which concluded that cannabis may offer symptom relief, even if more high-quality, large-scale, and long-term studies are needed to clarify its therapeutic potential and risks (Volz et al. 2016; Martín-Sánchez et al. 2009). This is particularly relevant for chronic pancreatitis, where pain management is a significant challenge.

The form of choice for the cannabis treatment of chronic pain is the one based on oils prepared with standardized extracts, whose exact dosages of CBD and THC are guaranteed. Cannabis therapy is characterized by a cautious and personalized approach. It always starts from very low dosages, as in the clinical case presented here, gradually increasing the dose until the desired therapeutic effect is achieved, minimizing side effects. A doctor might start with a low concentration oil (for example, a 5% CBD/THC oil), initially prescribing 2 drops, 2–3 times a day, considering that each drop of a 5% oil corresponds to about 2.5 mg of active ingredient, and increase the dose by 1 drop every 3–7 days, depending on the patient's response and tolerability.

A crucial aspect of medical cannabis is that there is no "ceiling effect" for its analgesic potential, in the sense that there is no maximum established dose beyond which no benefit is observed. This means that titration can continue until the effective dose tolerated by the patient is found. Inhalation administration (through specific medical vaporizers) is generally reserved for conditions that require rapid and immediate action, such as severe neuropathic pain, sudden pain ("breakthrough pain") or acute spasticity. The advantage is the onset of the effect in a few minutes, but the duration is shorter than oral intake. On the contrary, in Italy, resins or edible products such as cannabis-based gummy candies are not reimbursed by the National Health Service (SSN) and are not commonly used in medical therapies due to their variability in dosage.

Despite the potential benefits, it is important to consider the adverse effects associated with cannabis use. It has been reported that cannabis-based medicines increased the incidence of nervous system and psychiatric disorders, emphasizing the need for careful patient selection and monitoring (Mücke et al. 2018). This is crucial in ensuring that the benefits of cannabis treatment outweigh the risks, especially in patients with chronic conditions. According to available literature, cannabis-based therapies are generally well-tolerated in patients without specific contraindications. Most adverse effects are mild, transient, and primarily associated with THC, with their occurrence being dose-dependent (Mick and Douek 2024). The extract prescribed to the patient contains a high concentration of CBD (5%) and a low concentration of THC (< 0.5%), administered at minimal dosages. Consequently, the absence of adverse drug reactions (ADRs) since the initiation of treatment was unsurprising to the medical team.

Regarding the potential mechanism of action, cannabinoids such as CBD and THC exert their therapeutic effects in pancreatitis through several mechanisms. CBD has been shown to have significant anti-inflammatory properties. According to Li et al. (2013), CBD reduces inflammation in experimental acute pancreatitis by improving the expression of GPR55 in pancreatic tissue and decreasing pro-inflammatory cytokines like IL-6 and TNF-α (Li et al. 2013). Additionally, CBD modulates oxidative stress, a key factor in the pathogenesis of chronic pancreatitis, by reducing the levels of oxidative markers and increasing the expression of cytoprotective factors such as (Yong-yu (n.d.)). CBD also interacts with the adenosine A2A receptor, which plays a crucial role in mediating its anti-inflammatory effects. CBD's inhibition of adenosine uptake enhances adenosine signaling, leading to reduced inflammation and tissue damage in models of acute lung injury, which can be extrapolated to pancreatic inflammation (Ribeiro et al. 2012). This suggests that CBD's anti-inflammatory effects in pancreatitis are partly mediated by its interaction with the adenosine system. Moreover, cannabinoids inhibit pain signaling by acting on central and peripheral receptors, providing an analgesic effect that is beneficial in chronic pain conditions like pancreatitis. Indeed, activation of cannabinoid receptors CB1 and CB2 reduced pain and inflammation in acute pancreatitis without producing central adverse effects (Michalski et al. 2007). This indicates that cannabinoids can effectively manage pain while also addressing inflammation. Nonetheless, the presented case has several limitations. First, it represents a single-patient report, and the observed benefits cannot be generalized without further evidence from larger, controlled studies; furthermore, the patient's long clinical history must be taken into consideration, as she visited many different hospitals and laboratories, even in different countries, which makes it more difficult to reconstruct in detail all the treatment attempts made. Second, while the absence of adverse drug reactions in this patient may be attributed to the high CBD-to-THC ratio and minimal dosages used, these findings should be interpreted cautiously. Further research is needed to validate the therapeutic potential of cannabinoids in chronic pancreatitis and to establish a clear risk–benefit profile.

## Conclusion

This case illustrates the potential of medical cannabis as an effective treatment option for chronic, treatment-resistant pancreatitis, a condition notoriously difficult to manage with conventional therapies. The patient’s experience demonstrates how cannabinoids can provide substantial pain relief, reduce inflammation, and improve quality of life, even when standard interventions fail to yield lasting benefits. Her case underscores the importance of exploring alternative therapies for complex, chronic conditions like pancreatitis, suggesting that medical cannabis may offer a transformative option for patients with few viable treatment paths.

## Data Availability

The dataset supporting the conclusions of this article are not publicly available due to privacy reasons, but are available from the authors FAS (email to: feliceantonio.spaccavento@asl.bari.it) and/or to CDVS (email to: devirgiliocesare@gmail.com) upon reasonable request.
